# Intra-natal Torsion of Polydactyly

**DOI:** 10.4103/0974-2077.79197

**Published:** 2011

**Authors:** Sanjay Saraf

**Affiliations:** *Department of Plastic Surgery, NMC Specialty Hospital, Dubai, UAE*

**Keywords:** Polydactyly, polydactyly gangrene, polydactyly torsion, post-axial

## Abstract

Post-axial polydactyly is a common congenital hand anomaly with a wide range of manifestations. We report here an unusual case of intra-natal torsion of duplicated small finger which presented as gangrene at birth.

## INTRODUCTION

The duplication of the little finger is one of the most frequent congenital malformations of the hand. The extent of duplication varies from a soft tissue nubbin to a completely developed digit. The presented case probably represents the first reported case of intra-natal torsion of post-axial polydactyly presenting as gangrene at birth, as an extensive review of the literature and Pubmed search did not reveal a similar published case.

## CASE REPORT

**Figure 1:**
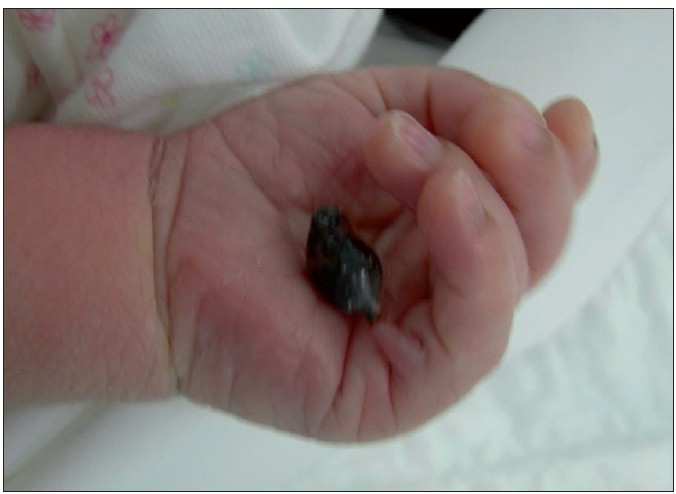
Torsion of polydactyly

**Figure 2:**
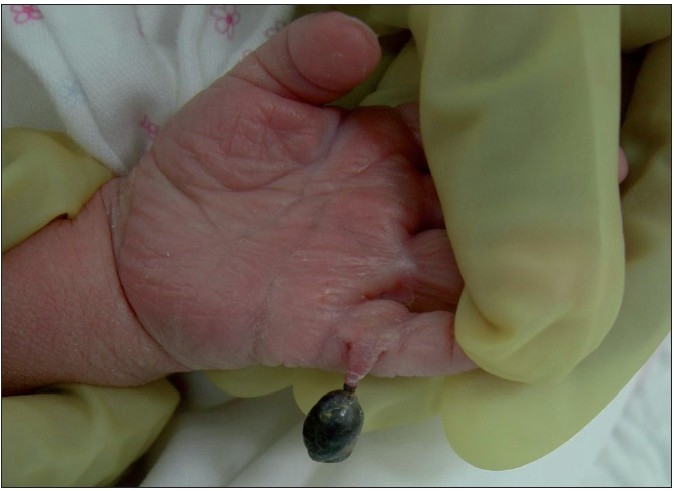
Torsion of polydactyly

**Figure 3:**
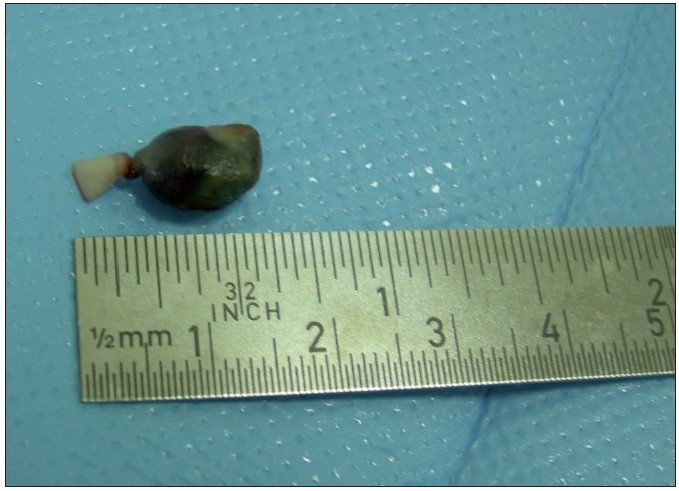
After excision

A male child presented with gangrene of the accessory digit of the left hand at birth [Figure 1]. The child’s weight was 6.25 kg and was delivered full term by Caesarean section. There was no similar or other obvious visible anomaly. There was no sibling or family history of any hand/feet or other congenital anomalies. The child was evaluated by a neonatologist and no associated cardiovascular, gastrointestinal or genitourinary system anomaly was found. The ultrasonography of abdomen was normal. The antenatal history was not significant in relation to exposure to viral infection, drugs, alcohol, or radiation in the first trimester. On examination, there was a gangrene of twisted pedunculated accessory digit of left little finger [Figure 2]. The gangrenous digit was excised from the base under local anaesthesia with satisfactory outcome [Figure 3].

## Discussion

Post-axial polydactyly constitutes one of the most frequent congenital malformations of the hand and is thought to be the result of an excess longitudinal segmentation representing probably an increased folding of the apical ectodermal ridge. The true incidence of post-axial polydactyly is difficult to determine as many of them are removed by the obstetricians and paediatricians in the nursery. Post-axial polydactyly occurs in approximately 1 in 300 black births and 1 in 3000 in Caucasians and is more frequently seen in male children.[1,2]

The spectrum of the deformity in post-axial polydactyly ranges from a simple soft-tissue problem to a complex completely developed accessory ray. Post-axial polydactyly has been classified into three types based on the degree of duplication.[3] Type I is duplication of soft parts only; Type II is partial duplication of the digit, including the osseous structures; and Type III is complete duplication of the digit, including the metacarpal, but is rare.

The torsion of pedunculated accessory digit is sometimes seen in Type I duplication, however, intra-natal torsion of post-axial polydactyly has not been previously reported in the literature to the best of our knowledge. The intra-natal torsion of visceral or parietal organs per se is very rare. An extensive search of the literature in Pubmed revealed only one case of intra-natal torsion of a testicular tumour.[4]

Post-axial polydactyly is believed to be genetically determined. The Type I pattern is dominant and multifactorial and involves two genes with incomplete penetrance. Type II and III are inherited as dominant traits with marked penetrance.[5] The black children usually present with autosomal dominant transmission with no other anomalies, while in Caucasians the transmission is autosomal recessive with frequent syndromic association.[5] The syndromes known to be associated with post-axial polydactyly are chromosomal syndromes (trisomy 13,18), bony dysplasias (Ellis van creveld syndrome, Achrondroplasia), syndromes involving eyes (Laurence moon biedl), syndromes involving skin (Goltz, Bloom), orofacial syndromes (cleft lip, Meckel) and syndromes with mental retardation (Cornelia de Lange, Smith-Lemli-Opitz).[6]

Post-axial polydactyly requires detailed clinical, syndromic and imaging evaluation prior to the treatment. The treatment modalities vary from simple excision to complex skeletal surgeries depending upon the type. For Type I deformities, early excision followed by primary closure and occasionally z-plasty closure (if the skin incision is in a palmar location) under local anaesthesia is the preferred treatment.[5,6] The use of only ligature around the base is not recommended because of reports of fatal haemorrhage.[5–7] The suture ligation at the base of the pedicle often leaves small nubbins with retained cartilage which later grow requiring subsequent removal.[6] The pedicle should be incised and the neurovascular pedicle should be cauterized or ligated directly.[8] The torsion of the pedunculated accessory digit invariably leads to pain, infection and occasionally significant bleeding. They should be considered as surgical emergency and should be excised immediately. The management of Type II and Type III is more complex and it is preferable to wait for at least two to three years as by this time the anaesthesia risks recede, the hand is larger and technically surgery is easier to perform. In Type III, more functional digit should be retained combined with the parts of the ablated partner, with placement in a more satisfactory position.[9]
